# Complete Genome Sequence of Yersinia ruckeri Strain CSF007-82, Etiologic Agent of Red Mouth Disease in Salmonid Fish

**DOI:** 10.1128/genomeA.01491-14

**Published:** 2015-01-29

**Authors:** Michael C. Nelson, Scott E. LaPatra, Timothy J. Welch, Joerg Graf

**Affiliations:** a Department of Molecular and Cell Biology, University of Connecticut, Storrs, Connecticut, USA; b Clear Spring Foods, Inc., Research Division, Buhl, Idaho, USA; c National Center for Cool and Cold Water Aquaculture, Agricultural Research Service, U.S. Department of Agriculture, Kearneysville, West Virginia, USA

## Abstract

We present the complete, closed, and finished chromosomal and extrachromosomal genome sequences of Yersinia ruckeri strain CSF007-82, the etiologic agent of enteric red mouth disease in salmonid fish. The chromosome is 3,799,036 bp with a G+C content of 47.5% and encodes 3,530 predicted coding sequences (CDS), 7 ribosomal operons, and 80 tRNAs.

## GENOME ANNOUNCEMENT

Yersinia ruckeri, a Gram-negative gammaproteobacterium, is the etiologic agent of enteric red mouth disease (ERM) a hemorrhagic septicemia of farmed salmonid fish species worldwide (1, 2). Y. ruckeri strains include several O-serotypes, yet nearly all outbreaks are caused by a genetically homogenous group of serotype 01 strains. Successful bacterin vaccines for ERM were developed in the 1970s; however, outbreaks in vaccinated fish have recently occurred and have been associated with the emergence of novel variants of this pathogen (3, 4). The reemergence of Y. ruckeri mediated ERM has renewed interest in the virulence and evolution of this pathogen.

Presently, only two draft Y. ruckeri genomes are available; the genome of the type strain, ATCC 29473^T^, consists of 174 contigs, while the genome of a strain from Chile consists of 75 contigs (5). An Illumina-based assembly of the pathogenic Y. ruckeri strain CSF007-82 (6) yielded 63 contigs with *N*_50_ of 160,889 bp. Because of this, we resequenced strain CSF007-82 using PacBio in order to obtain a complete, circularized genomic reference for use in future studies examining determinants of Y. ruckeri pathogenicity.

Large insert libraries were prepared and sequenced on a Pacific Biosciences RS II instrument using the P5-C3 sequencing chemistry at the Yale Center for Genomic Analysis, generating over 1.64 Gbp of sequencing data from ~200,000 raw reads. The resulting data was assembled using the HGAP version 2 assembler (7) with standard parameters, yielding two unique contigs ~100 kbp and ~3.8 Mbp in size. Overlapping nucleotides were manually edited and removed from the contig ends to create circularized replicons, which were then used as reference sequences for remapping of the raw sequencing reads to identify and correct any remaining errors. Interestingly, a contig corresponding to the plasmid pYR2 identified in the Illumina assembly was not generated, however, sequencing reads did map to pYR2 during the reference assembly. The largest contig, representing the chromosome, is 3,799,036 bp with a G+C content of 47.5% while the second contig is 103,917 bp with a G+C content of 48.4%. pYR2 is 16,923 bp with a G+C content of 47.2%.

The three finished contigs were functionally annotated by RAST (8). The chromosome encodes 3,517 predicted coding sequences (CDS), 22 rRNAs organized into 7 ribosomal operons, and 80 tRNAs covering all standard amino acids. Numerous phage and transposable element proteins were identified. The ~100 kbp contig encoded 99 predicted CDS, 16 of which belonged to the IncI1 class of replication proteins. No RNAs were predicted for this contig, indicating that it represents a previously unknown plasmid we named pYR3.

The availability of a complete, finished Y. ruckeri genome sequence, in combination with additional genome sequences should allow for more thorough analysis and identification of novel virulence factors associated with ERM. Additionally, the availability of a closed genome sequence should enable further studies of the evolution of the Yersinia genus as well as an examination of the transmission and acquisition of mobile genetic elements.

### Nucleotide sequence accession numbers.

The sequencing data is available under the GenBank/ENA/DDBJ accession no. PRJEB6967. The annotated genome is available under accession numbers LN681229 to LN681231.
